# Distance-construal relationship: Mediating role of perceived control and moderating role of locus of control

**DOI:** 10.3389/fpsyg.2022.975417

**Published:** 2023-01-30

**Authors:** Hui-dan Huang, Qi Zhang

**Affiliations:** ^1^School of Continuing Education, Nanjing University of Science and Technology, Nanjing, Jiangsu, China; ^2^School of Management, Zhejiang Gongshang University Hangzhou College of Commerce, Hangzhou, Zhejiang, China

**Keywords:** psychological distance, construal level, perceived control, locus of control, construal level theory

## Abstract

The construal level theory (CLT) has been supported and applied widely in social psychology. Yet, what remains unclear is the mechanism behind it. The authors extend the current literature by hypothesizing that perceived control mediates and locus of control (LOC) moderates the effect of psychological distance on the construal level. Four experimental studies were conducted. The results indicate that individuals perceive low (vs. high) situational control from a psychological distance (vs. proximity), and the resultant control perception influences their motivation in control pursuit, producing a high (vs. low) construal level. Moreover, LOC (i.e., one’s chronic control belief) affects an individual’s motivation to pursue control and yields a reversal of distance-construal relationship under external (vs. internal) LOC as a result. Overall, this research first identifies perceived control as a closer predictor of construal level, and the findings are expected to help with influencing human behavior by facilitating individuals’ construal level *via* control-related constructs.

## Introduction

Construal level (CL) reflects mental representation regarding cognitive targets. At high CL, people tend to focus on core, causal, abstract, structured, and decontextualized information; conversely, at low CL, people tend to focus on surficial, secondary, irrelevant, concrete, and contextualized information (Liberman and Trope, 1998; Trope and Liberman, 2010). Therefore, people’s prediction, evaluation, and behavior change accordingly with CL. Existing studies show that psychological distance from the target is a central predictor of CL in that people construe at a low (vs. high) level when it is psychologically proximal (vs. distant), and vice versa, forming construal level theory (CLT) (Liberman and Trope, 1998; Trope and Liberman, 2010).

However, the phenomena have seen exceptions that individuals construe at a low (vs. high) level when being psychologically distant (vs. proximal). For example, a new graduate might give up a job interview arranged a month later due to transporting and scheduling inconvenience that inhibits feasibility, instead of valuing it in terms of job desirability. Moreover, when concerning environmental and pandemic context, psychological distance sometimes cannot be easily altered in a short time, yet it is still necessary to guide people to think at a certain CL to avoid negative consequences, such as anxiety and despair. For example, encountering the COVID-19 epidemic, in order to avoid national panic risk in China, appropriate policies aimed at evoking high CL should be made so that people can cognize COVID-19 pandemic in a more rational and global way, in spite of the unchangeable psychological proximity. These above exceptions go against the prediction of CLT, and it raises questions as to how psychological distance exactly influences CL and what is the boundary factor that leads to the reversal of CLT prediction.

These questions are addressed by introducing a closer predictor of CL that follows after psychological distance (i.e., perceived control) and a moderator [i.e., locus of control (LOC)]. The research uncovers a control-based route to explain the relationship between psychological distance and CL (distance-construal relationship). It is argued that psychological distance decreases control perception and motivates individuals to regain control, leading to an abstract level of thinking to dominate. Furthermore, it is proposed that the distance-construal relationship is dependent upon the LOC. When one’s LOC is external (vs. internal), psychological distance (vs. proximity) leads to a low (vs. high) construal level, reversing the CLT prediction.

## Psychological distance and perceived control

Psychological distance is a subjective experience that the cognitive target is close or far away from the self, here, now, and certainty (Trope and Liberman, 2010), with four dimensions of psychological distance, namely, temporal, social, spatial, and hypothetical distances (Trope and Liberman, 2010). Control is a primary driver of human behavior and a basic need for human survival (Miller, 1979). Perceived control is defined here to be how much control people feel they have when facing one specific situation; hence, it is focused on the control that is highly contingent upon situations. There exist two ways of operationalization for perceived control. The first way points out that perceived control is the sense of control people perceive and feel to have in terms of choice, predictability, responsibility, and ability to reduce or get relief from unpleasant situations (Averill, 1973; Baronas and Louis, 1988). The second operationalization includes personal mastery and perceived constraints (Su et al., 2017). Personal mastery is the extent to which people perceive themselves to influence or control the environment, and perceived constraints are the extent to which people believe there are obstacles beyond their control that interfere with their goals (Gurin et al., 1978; Lachman and Weaver, 1998).

In light of definitions and lay theory, psychological distance is proposed to be negatively related to perceived control. According to control’s first operationalization, being distant makes it harder to ascertain the choices people make. Compared with predicting what will happen for remote relatives a year later, it is easier to predict that of tomorrow for oneself. Furthermore, changes one makes at one moment reveal consequences now and here for sure, while the consequential effect disappears over time and across spaces. According to the second operationalization, being distant (vs. proximal) from here, now, self, and certainty indicates loss or unavailability of contextual and relevant information, and it is consequently associated with the inability to influence the environment effectively, impairing one’s personal mastery. Likewise, distance (vs. proximity) puts a limit on one’s apprehension of the environment and interactions that are far away, resulting in more (vs. less) perceived constraints. Taken together, it is natural to postulate that psychological distance (vs. proximity) makes people perceive a lower (vs. higher) sense of control.

Adding to the above argument, other supportive empirical literature in behavioral science has implicitly provided preliminary evidence for a distance-control link. For example, people being spatially proximal with cognitive targets perceive higher control when introducing computer-based information systems within corporations (Baronas and Louis, 1988). General social exclusion reduces the sense of control (Su et al., 2017) since being socially distant and rejected by others impairs one’s personal mastery over surroundings and results in more perceived constraints due to their limitations in societal understanding (Bruneau, 1973; Skinner, 1996). When on a social network, both higher inhabited space and higher isomorphic effects in social media such as YouTube can not only make users feel more controllable in terms of real time, context, and interaction but also reduce users’ psychological distance (Lim et al., 2012), suggesting a negative link between distance and control.

Therefore, the above demonstrations converge on the argument that psychological distance (vs. proximity) leads to lower (vs. higher) perceived control.

## Perceived control and construal level

When being psychologically proximal, one perceives a low sense of control and naturally desires to restore it (Fiske et al., 1996). The need for control restoration is grounded in two theories. First, psychological reactance theory (Wortman and Brehm, 1975) postulates that an unfulfilled need for control creates discomfort and tension, prompting subsequent reactions to augment perceived control. Second, control motivation theory (Pittman and D’Agostino, 1989) claims that when there is a discrepancy between the current and desired state, one would be motivated to reduce it (Locke and Latham, 2002), resulting in control compensation when perceived control is low and desired control is high. Therefore, it is asserted that when perceiving lower control due to psychological distance, one is motivated to restore control.

People might restore control by working directly on sources of control deprivation. However, the sources mostly do not seem to be amendable. Concerning this present research, the uncontrollability caused by psychological distance usually cannot be restored by being in charge of specific behaviors because distant cognitive targets are mostly inaccessible and unavailable within the traditional five sensory systems. In other words, experiencing psychological distance, in order to restore control, one cannot execute specific behaviors on targets by construing at a lower level that focuses on surroundings. Hence, people would choose alternatives to compensate control indirectly.

One way to compensate control is to utilize specific mindsets, including problem-solving (Su et al., 2017) and structure-seeking (Kay et al., 2008; Whitson and Galinsky, 2008) mindsets. Structure-seeking mindset is more relevant in this study because high-level construal by nature dictates structured and simple-patterned mental representation, providing a sense of structure to regain control. Preliminary evidence can be learned from prior studies that adopt categorization tasks to measure CL. Specifically, categorization breadth is broader under high CL (Trope and Liberman, 2010), verifying that people construing at high CL tend to form illusionary patterns regarding random and various items. The pattern is considered identical to the structure in this study. Hence, it is reasonable to postulate that high-level construal can be broadly considered as one type of structure-seeking mindset, helping restore control.

Moreover, extant empirical studies have shown that control deprivation exerts consequential effects on information processing and thinking style that are consistent with features of high-level construal. People deprived of control would process available information diagnostically (Swann et al., 1981), deliberately (Pittman and Pittman, 1980; Gollwitzer, 2003), and analytically (Zhou et al., 2012), resulting in judgment preferences that are at high CL. Precisely, diagnostic thinking heightens one’s interest in central and potentially essential information; deliberate thinking allows one to accurately assess desired outcome (Gollwitzer and Kinney, 1989) with a focus on the desirability; analytical thinking enables people to process *via* systematic observation to deduce general, stable, and abstract laws or principles that are beneficial to more accurate predictability (Zhou et al., 2012), all above matching with tenets and features of high CL (Liberman and Trope, 2014). Furthermore, control-deprived individuals are more likely to make attributional and causal analyses, and this cause-focus (vs. effect-focus) is exactly another feature representing high CL (Liberman and Trope, 2014).

When being psychologically proximal, differently, the consequential high control perception results in a feeling of overconfidence (Jewell and Kidwell, 2005; Stotz and Von Nitzsch, 2005; Peluso et al., 2017). Being overconfident, one would be discouraged to engage significant cognitive resources because they are complacent with current states and feel a substantial low gap between their current and desired states (Jewell and Kidwell, 2005). Similarly, according to goal and motivation literature (Fishbach and Dhar, 2005; Wilcox et al., 2009), when individuals have the opportunity to perceive higher control that is consistent with their natural desire, this in turn temporarily licenses them to indulge themselves to release motivation for control and quit investing effort on control pursuit. Yet, effortful reflection and cognitive resource utilization are essential for high CL and required for thinking styles like deliberate processing and reasoning analyses (Fazio, 1990; Jewell and Kidwell, 2005). When people do not intend to control, it is reasonable to speculate they would process portions and peripheral details from surroundings, construing at a low level subsequently. Thus, overconfidence leads to judgments that are focused on details, effects, means, and situations, matching features of low CL (Liberman and Trope, 2014).

Therefore, the above demonstrations converge on arguments that low perceived control predicts high-level construal and high perceived control predicts low-level construal. It is due to the compensating role of high CL for control loss, the effect of low control perception on information processing and thinking style, as well as the close linkage between high control and overconfidence.

Put all together, it is claimed that psychological distance influences construal level *via* perceived control. Then, another question would be raised as to what factor may influence this underlying control-based mechanism route. In other words, what is the boundary? Next, it is argued that the LOC moderates the distance-construal relationship by influencing the motivation related to control pursuit.

## Reversal of distance-construal relationship

Although psychological distance (vs. proximity) leads to lower (vs. higher) control perception situationally, one’s chronic control belief cannot be easily altered. Regarding this current research, two cognitive orientations regarding control beliefs matter for the distance-construal link, and they are the internal and external LOC. Unlike perceived control from specific situations, the LOC describes trait-like individual differences in the chronic tendency to perceive oneself as having control and attempting to control their personal environment in general (Williams et al., 2004), remaining stable across situations and forms of action (Ajzen, 1991). People with internal LOC believe that environmental events and outcomes are contingent upon their own actions, while people with external LOC believe that external factors such as chance, fate, or powerful others control outcomes (Rotter, 1966; Zhou et al., 2012). Therefore, when one’s LOC is internal, as the abovementioned theorizing, psychological distance (vs. proximity) leads to lower (vs. higher) perceived control and then motivates (vs. discourages) people to establish control by construing at a high level. Yet, when one’s LOC is external, it is expected that the distance-construal relationship would reverse.

Confronting with control deprivation due to psychological distance, people with external LOC would adjust themselves passively to fit the environment and cease the motivation to restore their own control (Wortman and Brehm, 1975). They think they will not master the situation whatever they do (Ric and Scharnitzky, 2003) and are more likely to be trapped into learned helplessness (a conditioned response to control deprivation) (Abramson et al., 1978), leading to effort reduction or give-ups. People will no longer be willing to engage in effortful cognitive activities (Ric and Scharnitzky, 2003), activating their low CL. Therefore, it is predicted that when LOC is external, even though control perception is low after experiencing psychological distance, people tend to quit turning to abstract rules, stable principles, and consistent laws to restore control, construing at a low level eventually. Conversely, psychological proximity produces high control, and individuals’ state LOC would be temporarily stimulated to be internal, dominating their trait external LOC. Subsequently, people would be motivated to seek more control due to the reinforcement effect (Rotter, 1966). Based on the inference about the compensating role of high CL for control, it can be sufficiently stated that, under psychological proximity conditions, temporary internal state LOC successively motivates people to reassert seeking control and construe at a high level as a result.

## Research overview

**FIGURE 1:**
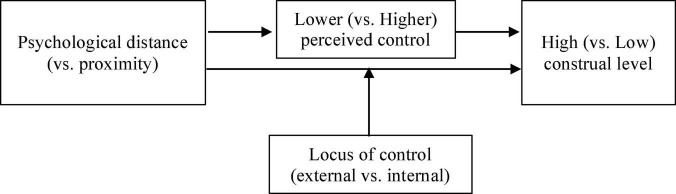
Theoretical model.

To summarize the abovementioned theorizing conjunctly, four experimental studies were designed to test the following hypotheses (see Figure 1). First, the effect of psychological distance on the construal level is mediated, at least in part, by perceived control. Specifically, psychological distance (vs. proximity) predicts lower (vs. higher) control perception, and lower (vs. higher) control perception yields high-level (vs. low-level) construal due to the compensating role of high (vs. low) CL for control. Second, LOC moderates the distance-construal relationship. Specifically, the distance-construal relationship maintains when one believes in internal LOC; yet, the construal consequences of psychological distance reverse when one believes in external LOC.

Study 1a focused on control perception changes and control-restoring needs that result from psychological distance. Study 1b aimed to show that high (vs. low) CL indeed increases the sense of control, explaining why low (vs. high) control perception triggers high (vs. low) CL. These two studies verified the premises that are important for hypothesis development, providing justification to continue formal experimental studies to test the hypotheses of the mediation role of perceived control and moderating role of LOC. Study 2 directly testified that the distance-construal relationship is mediated by perceived control. Study 3 showed that the distance-construal relationship holds under internal LOC and instead reverses under external LOC.

Across studies, the psychological distance was manipulated in three ways, namely, spatial, hypothetical, and social distances, to improve validity. All manipulating procedures were adopted from prior literature. Both indirect and direct measurements of CL were applied in that CL was separately operationalized as the breadth of categorization in study 1b and measured using the Behavioral Identification Form (BIF) (Vallacher and Wegner, 1989) in study 3. Sample sizes were determined based on similar CLT studies (Liberman et al., 2002; Brügger et al., 2016). Because climate change text material was developed in the United States context in study 1a, only participants who resided in the United States were recruited. Except for this condition, no limits on other demographic information were put for all potential participants. All studies adhered to ethical guidelines specified in the APA code of conduct as well as the authors’ national ethics guidelines. All studies collected data one by one in sequence, and individuals who already had participated in one study were not allowed to participate in another. It is assured that all the participants appeared only once in all studies, improving sample diversity and avoiding bias from practice effect. All measures and manipulations are reported. To ensure that all manipulations were randomly distributed, thus establishing the internal validity of all studies, balancing tests were conducted. The results showed that there were no statistically significant differences between conditions on any of the demographic variables for all studies, and therefore, the randomization was successful (see Table 1). The demographic characteristics of all studies are reported in Table 2. In all studies, standard procedures were followed to exclude participants who skipped manipulations, failed manipulation-check questions, were suspicious about study objectives, and/or explicitly reported difficulties in understanding instructions and materials. Data were analyzed without adding or excluding on purpose.

**TABLE 1:** Balancing test results for all studies.

Demographic variables	Study 1a		Study 1b		Study 2		Study 3	
	*F*(1, 123)	*p*	*F*(1, 184)	*p*	*F*(1, 102)	*p*	*F*(1, 188)	*p*
Sex	0.199	0.656	0.091	0.763	0.955	0.331	0.210	0.647
Age	0.336	0.563	0.885	0.348	2.727	0.102	2.816	0.095
Education	0.077	0.782	0.124	0.725	/	/	/	/
Wage	0.324	0.570	0.651	0.421	/	/	/	/

Student participants were recruited in study 2 and study 3, so data on education and wage level regarding these two studies do not apply.

**TABLE 2:** Demographic characteristics of all studies.

Demographic variables	Study 1a		Study 1b		Study 2		Study 3	
	Mean	SD	Mean	SD	Mean	SD	Mean	SD
Sex	1.51	0.502	1.52	0.501	1.53	0.502	1.52	0.501
Age	33.59	12.398	32.55	12.299	19.88	1.797	20.11	1.703
Education	1.92	0.617	1.93	0.682	/	/	/	/
Wage	2.46	1.012	2.26	0.996	/	/	/	/

Student participants were recruited in study 2 and study 3, so data on education and wage level regarding these two studies do not apply.

## Study 1a

Study 1a examined whether psychological distance (vs. proximity) reduces individuals’ sense of control and motivates them to regain control. Results would provide a partial reason to examine the mediating role of perceived control in study 2.

### Procedure

In total, 125 participants (51.2% female; *M*_age_ = 33.59, *SD* = 12.40) were recruited for a fee through the Prolific platform. A sensitivity analysis using G*power (Faul et al., 2007) showed that this sample size could test medium-to-large effect sizes of *f* = 0.32 and *d* = 0.64 for α = 0.05 and 1-β = 0.95 levels.

Prior studies have highlighted a high need for structure after control loss (Cutright et al., 2013) and that a structure-seeking mindset compensates control (Kay et al., 2008; Whitson and Galinsky, 2008; Cutright, 2012). Therefore, the need for structure (Neuberg and Newsom, 1993) was utilized as a proxy to measure motivation to restore control.

Differences in participants’ perceived control and need for structure were examined using one factor (spatial distance: proximal vs. distant) between-subjects design. Eligible participants were required to reside in the United States and were randomly assigned to both conditions. Adopting Brügger et al. (2016), in proximal condition, participants were instructed to read texts about the impacts of climate changes in the United States, and location words were mentioned 13 times. In distant condition, participants were instructed to read texts about the impacts of climate changes worldwide, and location words were also mentioned 13 times. For both conditions, the texts were exactly the same except for those bold location words.

Then, psychological distance, perceived control, and need for structure were assessed separately. Adopting prior research (Van Boven et al., 2010; Brügger et al., 2016), participants were asked to indicate their psychological distance using a five-item 7-point scale: “To me, climate change feels very close … very distant; To me, climate change feels like here … like at the other end of the world; To me, climate change feels like tomorrow … like thousands of years away; To me, climate change feels like affecting me … like affecting distant strangers; To me, climate change feels very real … very hypothetical”. These items were averaged to form a psychological distance index (α = 0.76). Adopting Armitage et al. (1999), perceived control was assessed using a four-item 7-point scale that asked participants to indicate their thoughts on the following statements: “I believe I have the ability to confront with climate change (1 = strongly disagree, 7 = strongly agree); To what extent do you see yourself as being capable of governing climate change (1 = very incapable, 7 = very capable); If it were entirely up to me, I am confident that I would be able to solve climate change issue (1 = strongly disagree, 7 = strongly agree); How confident are you in your ability to work effectively on climate change issue? (1 = not at all confident, 7 = definitely confident).” These items were averaged to form a perceived control index (α = 0.85). Lastly, adopting the scale of need for structure from Neuberg and Newsom (1993), participants were asked to indicate their agreement (1 = strongly disagree, 7 = strongly agree) with 11 statement items (e.g., I enjoy having a clear and structured mode of life), and these items were averaged to form the need for structure index (α = 0.74). Participants then reported their demographic information.

### Results

#### Manipulation check

Independent sample *t*-tests showed that the psychological distance index in spatially proximal (vs. distant) condition was significantly lower (*M*_proximal_ = 2.37, *SD* = 0.64; *M*_distant_ = 3.51, *SD* = 1.48; *t* = −5.61, *p* < 0.001, *d* = 0.61; 95%CI: −0.8355, −0.3739), indicating successful manipulation.

#### Perceived control

**FIGURE 2:**
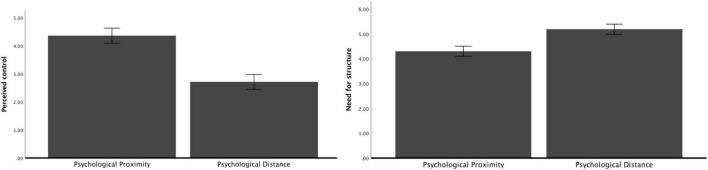
Perceived control and need for structure as a function of psychological distance (study 1a).

After controlling demographic characteristics, one-way ANOVA showed that participants perceived greater control in proximal (vs. distant) condition [*M*_proximal_ = 4.35, *SD* = 1.16; *M*_distant_ = 2.72, *SD* = 1.09; *F*(1, 123) = 73.83, *p* < 0.001, η^2^ = 0.38; 95%CI: 0.2436, 0.4833; see Figure 2].

#### Need for structure

After controlling demographic characteristics, one-way ANOVA showed that participants had a lower need for structure to compensate control in proximal (vs. distant) condition [*M*_proximal_ = 4.28, *SD* = 0.80; *M*_distant_ = 5.20, *SD* = 0.89; *F*(1, 123) = 37.19, *p* < 0.001, η^2^ = 0.24; 95%CI: 0.1127, 0.3492; see Figure 2].

### Discussion

Study 1a showed that psychological distance influences perceived control and the need for structure to compensate control in a way that perceived control is higher and the need for structure is lower when it is psychologically proximal (vs. distant). The results provide support for the prediction that psychological distance (vs. proximity) reduces individuals’ control perception and stimulates their desire to restore control subsequently.

## Study 1b

Stepping further, study 1b examined why perceived lower control and consequently motivation to restore control trigger abstract CL. It is speculated that high (vs. low) CL dictates mental representation that pursues structure and pattern, thus contributing to control compensation. In other words, the shift from low to high CL helps with control restoring. This notion is consistent with prior work that examined the instrumentality of thinking style in regaining control (Reuven-Magril et al., 2008). For example, Zhou et al. (2012) verified that analytical (vs. holistic) thinking helps to increase the sense of control. Similarly, it is predicted that participants primed with high (vs. low) CL would perceive higher control and show a lower need to restore control accordingly. Same as in study 1a, the need for structure was used as a proxy to measure the need to restore control.

### Procedure

In total, 186 participants (51.6% female; *M*_age_ = 32.55, *SD* = 12.30) were recruited through the Prolific platform for a fee. A sensitivity analysis using G*power (Faul et al., 2007) showed that this sample size could test medium-to-large effect sizes of *f* = 0.27 and *d* = 0.54 for α = 0.05 and 1-β = 0.95 levels.

Differences in participants’ perceived control and need for structure were examined using one factor (CL: low vs. high) between-subjects design. Adopted from Freitas et al. (2004), participants were randomly assigned to different conditions. In the low-CL condition, participants were instructed to describe how they would go about maintaining good personal relationships step by step on different survey pages following examples illustrated in the instruction section. The example went like this: “how can one move toward being happy?—by getting a good job.—how can one get a good job?—by earning a degree.—how can one earn a degree?—by completing course requirement.—how can one complete course requirement?—by communicating with instructors and mentors.” Likewise, in the high-CL condition, participants were instructed to describe why they would go about maintaining good personal relationships step by step on different survey pages following another example showcased. The example went like this: “why would you complete course requirement?—to earn a degree.—why would you earn a degree?—to develop and verify knowledge and skills.—why would you develop and verify knowledge and skills?—to get a good job.—why would you get a good job?—to have a happy life.”

After task priming, adopting Laran (2010), participants were asked to indicate whether maintaining good personal relationships for them was “connecting with, having party with, and sending gifts to family and friends,” anchored at 0 on the scale, or “the basis for a harmonious life,” anchored at 10 on the scale, forming as CL-check measurement. Then, the perceived control index (α = 0.92) and the need for structure index (α = 0.82) were measured using scales from study 1a except that they were adjusted to scenarios in study 1b. Then, participants reported their demographic information.

### Results

#### Manipulation check

Independent sample *t*-test showed that CL-check score in high-CL condition was significantly higher than that in low-CL condition (*M*_high_ = 7.84, *SD* = 2.19; *M*_low_ = 4.69, *SD* = 1.39; *t* = −11.65, *p* < 0.001, *d* = 0.95; 95%CI: −1.1354, −0.7525), indicating successful manipulation.

#### Perceived control

**FIGURE 3:**
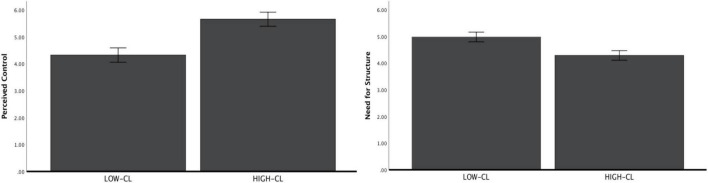
Perceived control and need for structure as a function of construal level (study 1b).

After controlling demographic characteristics, one-way ANOVA showed that participants perceived greater control in high-CL (vs. low-CL) condition [*M*_high_ = 5.66, *SD* = 0.82; *M*_low_ = 4.32, *SD* = 1.64; *F*(1, 184) = 49.38, *p* < 0.001, η^2^ = 0.22; 95%CI: 0.1156, 0.3083; see Figure 3].

#### Need for structure

After controlling demographic characteristics, one-way ANOVA showed that participants had a lower need for control restoring in high-CL (vs. low-CL) condition [*M*_high_ = 4.29, *SD* = 0.97; *M*_low_ = 4.96, *SD* = 0.79; *F*(1, 184) = 28.31, *p* < 0.001, η^2^ = 0.14; 95%CI: 0.0547, 0.2250; see Figure 3].

### Discussion

Study 1b showed that perceived control is higher and the need for structure is lower when participants construe at high (vs. low) level, proving that high CL helps with control restoring. Overall, the evidence supports the premise for the mediation prediction that psychological distance (vs. proximity) causes abstract (vs. concrete) CL through lower control perception and provides partial evidence to examine the mediating role of perceived control in study 2.

### Study 2

Study 2 sought to constructively replicate study 1 by manipulating hypothetical distance, and then, it investigated whether psychological distance influences CL *via* perceived control. It is expected that psychological distance (vs. proximity) leads to lower perceived control, and this control perception prompts participants to use abstract (vs. concrete) CL accordingly. Furthermore, this study also ruled out alternative accounts underlying CLT.

### Procedure

In total, 104 participants (52.9% female; *M*_age_ = 19.88, *SD* = 1.80) were recruited from an eastern university in China for course credit. A sensitivity analysis using G*power (Faul et al., 2007) showed that this sample size could test medium-to-large effect sizes of *f* = 0.36 and *d* = 0.72 for α = 0.05 and 1-β = 0.95 levels.

Mediating hypotheses were examined using one factor (hypothetical distance: proximal vs. distant) between-subjects design. Adopting Liberman et al. (2002), the hypothetical distance was manipulated using a scenario of going on a camping trip. The scenario was described as almost certain to occur (proximal condition) or almost certain not to occur (distant condition). Participants were randomly assigned to one of the two scenarios. They were asked to imagine planning to engage in the assigned activity. After scenario priming, the manipulation was checked, and perceived control was measured using the scale from study 1a except that it was adjusted to scenarios in study 2. The scale items were averaged to form a perceived control index (α = 0.93).

Then, the categorization task was instructed. The participants were presented a list of 38 activity-related items and were asked to imagine planning to engage in the assigned activity and to place activity-related items into groups that they thought belonged to one category to the same box. Participants were reminded they can create groups as many as or as less as possible, provided that no items were excluded and no groups should overlap. For the dependent variable, the breadth of categorization would be one quantification for CL, in that broader (vs. narrower) categorization represents high (vs. low) CL. The breadth of categorization was measured directly using the number of groups. The fewer the number of groups, the broader the breadth of categorization, and the higher the CL. Then, participants reported their demographic information.

### Results

#### Manipulation check

An independent sample *t*-test showed that the psychological distance index in the distant condition was significantly higher than that in the proximal condition (*M*_distant_ = 5.96, *SD* = 1.36; *M*_proximal_ = 1.81, *SD* = 1.17; *t* = −16.70, *p* < 0.001, *d* = 1.65; 95%CI: −1.9410, −1.3471), indicating successful manipulation.

#### Perceived control

**FIGURE 4:**
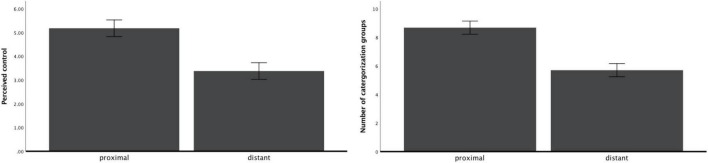
Perceived control and number of groups as a function of psychological distance (study 2).

After controlling demographic characteristics, one-way ANOVA showed that participants perceived greater control in proximal condition [*M*_proximal_ = 5.13, *SD* = 1.13; *M*_distant_ = 3.42, *SD* = 1.37; *F*(1, 102) = 50.47, *p* < 0.001, η^2^ = 0.34; 95%CI: 0.1877, 0.4529; see Figure 4].

#### Breadth of categorization

After controlling demographic characteristics, one-way ANOVA showed that participants in the distant condition grouped activity-related items with broader categorization, resulting in a smaller number of groups [*M*_distant_ = 5.71, *SD* = 1.58; *M*_proximal_ = 8.65, *SD* = 1.67; *F*(1, 102) = 79.78, *p* < 0.001, η^2^ = 0.45; 95%CI: 0.2955, 0.5479; see Figure 4].

#### Mediation analyses

To check whether the mediating role of perceived control is significant, mediation analysis with 5,000 bootstrapped samples was conducted (Hayes, 2013; Model 4). As expected, the conditional indirect effect of psychological distance (vs. proximity) on categorization breadth *via* perceived control was negative and significant (β = −0.60, *SE* = 0.25), with a 95% confidence interval (CI) excluding zero (CI: −1.1262 to −0.1600). Given that the total effect of psychological distance on categorization breadth is −2.94, 20.41% of the total effect is explained by perceived control. These results provided support for the theorizing that perceived control represents a motivational mediator under the distance-construal relationship.

### Discussion

One might argue that manipulation can influence participants’ mood and attention, causing emotion or attention effect toward our hypotheses. To rule out the effect of mood, participants were asked to complete the PANAS Scale (Watson et al., 1988; positive mood items: α = 0.91; negative mood items: α = 0.90) in the study. As for attention, two items from the PANAS Scale were averaged to form the attention index (α = 0.60): “To what extent do you feel alert right after reading the material above? To what extent do you feel attentive right after reading the material above?” Analyses showed that participants in both conditions did not differ significantly in their positive [*M*_distant_ = 3.77, *SD* = 1.24; *M*_proximal_ = 3.82, *SD* = 1.32; *F*(1, 102) = 0.08, *p* = 0.785] and negative mood [*M*_distant_ = 2.01, *SD* = 0.88; *M*_proximal_ = 1.82, *SD* = 0.92; *F*(1, 102) = 1.19, *p* = 0.277]. Additionally, participants did not differ in their attention in both conditions [*M*_distant_ = 4.04, *SD* = 1.42; *M*_proximal_ = 3.76, *SD* = 1.39; *F*(1, 102) = 1.20, *p* = 0.277]. All results ruled out the possible effects of emotion and attention.

### Study 3

Heretofore, this research is built upon the premise that perceived control is conceptualized under the realm of primary control and internal LOC (Zhou et al., 2012). However, due to chronic influences from cultural, contextual, and religious factors, people may hold the belief that they are not agents to determine outcomes and instead change themselves to suit the environment, practicing secondary control and external LOC. In line with theorizing, it is expected in study 3 that the distance-construal relationship only holds under internal LOC and reverses under external LOC. Moreover, this study was extended in its design by differently manipulating social distance and using the Behavioral Identification Form (BIF, Vallacher and Wegner, 1989) scale to measure CL.

### Procedure

In total, 190 participants (52.1% female; *M*_age_ = 20.11, *SD* = 1.70) were recruited from an eastern university in China for course credit. A sensitivity analysis using G*power (Faul et al., 2007) showed that this sample size could test medium-to-large effect sizes of *f* = 0.26 and *d* = 0.52 for α = 0.05 and 1-β = 0.95 levels.

The moderating hypothesis was examined using a 2 (social distance: distant vs. proximal) x 2 (LOC: internal vs. external) between-subjects design with BIF score as the dependent variable. After reading the introduction and giving consent, participants were first asked to fill out a survey to measure their inherent trait LOC. Following the study by Chaxel (2016), it was measured using a unidimensional scale (Rotter, 1966). There were 15 pairs of contradictory statements. For each pair, one statement reflected internal LOC (e.g., “People’s misfortunes result from the mistakes they make” scored as 1) and the other represented external LOC (e.g., “Many of the unhappy things in people’s lives are partly due to bad luck” scored as 0). Participants were instructed to select the statement they more strongly believed to be the case and not the one they would like to be true. The total score was calculated as a measurement of LOC, and the potential values ranged from 0 to 15, with lower (vs. higher) numbers indicating an external (vs. internal) LOC. Participants scoring below/above average were considered as external/internal group. After this section, a 1-min break was arranged to minimize the salience of the above measurement.

Then, following prior literature (Majid et al., 2007), sentence fragments that each described a metaphorical exchange interaction between two actors were used to manipulate social distance. Two actors were all third-person pronouns (e.g., Tom got a hug from Janet …) in socially distant condition, while these sentences were slightly modified such that one of the two actors’ names was changed to second-person pronoun—you (e.g., Tom got a hug from you …) in socially proximal condition. Participants were randomly assigned to each condition, and each participant was presented with 6 sentence fragments. They were told to imagine various events and come up with an appropriate continuation for each sentence. To ensure participants actually thought carefully, each sentence fragment remained on screen for a fixed 20-s duration before participants could submit their continuation and click “next.” After the task, social distance manipulation was checked using one question: “Please recall the above 6 sentence continuation and indicate to what extent these situations make you feel on a scale from 0 to 10, in which 0 is anchored “very close” and 10 is anchored “very distant.” Lastly, the BIF was used to assess CL. Then, participants reported their demographic information.

### Results

#### Manipulation check

Independent sample *t*-tests showed that the psychological distance in socially proximal (vs. distant) condition was significantly lower (*M*_proximal_ = 3.05, *SD* = 1.30; *M*_distant_ = 6.16, *SD* = 1.38; *t* = −15.95, *p* < 0.001, *d* = 1.17; 95%CI: −1.3514, −0.9795), indicating successful manipulation.

**FIGURE 5:**
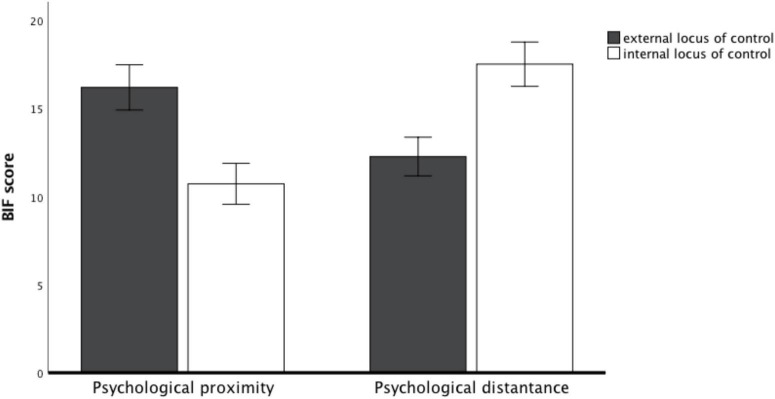
BIF score as a function of the interaction of psychological distance and locus of control (study 3).

*Behavioral Identification Form task*. After controlling demographic characteristics, two-way ANOVA was conducted, with psychological distance manipulation as the independent variable, external (vs. internal) LOC as the moderator, and BIF score as the dependent variable. The results revealed a significant main effect of psychological distance manipulation [*M*_distant_ = 14.55, *SD* = 5.17; *M*_proximal_ = 13.19, *SD* = 4.67; *F*(1, 186) = 5.94, *p* = 0.016, η^2^_p_ = 0.03; 95%CI: 0.0009, 0.0937] and an insignificant main effect of LOC [*M*_internal_ = 13.86, *SD* = 4.64; *M*_external_ = 13.93, *SD* = 5.29; *F*(1, 186) = 0.14, *p* = 0.709, η^2^_p_ = 0.001]. More importantly, consistent with theorizing, two-way interaction was significant [*F*(1, 186) = 74.37, *p* < 0.001, η^2^_p_ = 0.29; 95%CI: 0.1820, 0.3812; see Figure 5]. Planned contrast analyses showed that distance-construal prediction was reversed for the external LOC group. Specifically, socially proximal participants scored significantly higher on the BIF task than socially distant participants [*M*_distant_ = 12.27, *SD* = 5.19; *M*_proximal_ = 16.20, *SD* = 4.59; *F*(1, 186) = 21.01, *p* < 0.001, η^2^_p_ = 0.10; 95%CI: 0.0337, 0.1878]. However, this reversing effect disappeared under the internal LOC group in which socially distant (vs. proximal) participants scored significantly higher [*M*_distant_ = 17.51, *SD* = 3.33; *M*_proximal_ = 10.72, *SD* = 3.02; *F*(1, 186) = 61.36, *p* < 0.001, η^2^ = 0.25; 95%CI: 0.1478, 0.3445].

### Discussion

Study 3 provides support to the moderating theorizing of LOC, simultaneously providing indirect evidence for the mediating hypothesis regarding perceived control.

## General discussion

To conclude, this current research takes a motivational perspective to uncover the mechanisms underlying the distance-construal relationship by introducing the mediating role of perceived control and moderating role of LOC. Drawing on diverse literature and experimental studies, it illustrates that psychological distance alters people’s control perception in a way that they perceive high (vs. low) control for psychological proximity (vs. distance), and this results in low (vs. high) CL. Furthermore, LOC moderates the distance-construal relationship in a way that the distance-construal relationship holds under internal LOC and reverses under external LOC.

Across all studies, consistent evidence was found for the robust predictions. Study 1a established the starting point for later research with proof that psychological distance influences individuals’ control perception and their subsequent motivation to regain control. Study 1b found that different construal levels activate different control perceptions, displaying the functional role of high CL in control compensation. Study 2 presented a reliable test for the mediation role of perceived control, delivering evidence for the control-based mechanism underlying the distance-construal relationship. Finally, study 3 revealed a control-inspired boundary factor, LOC, that reverses the distance-construal relationship by exerting an effect on motivation to regain control, providing additional indirect evidence for the mediation assertion.

For CLT, the findings make three noteworthy contributions. First and foremost, the research adds to the growing literature on understanding the black box underlying CLT by verifying the mediating role of perceived control. The findings suggest that psychological distance (vs. proximity) is linked to low (vs. high) control perception and then motivates individuals to adopt high (vs. low) CL to make a response. This motivational approach serves as a complementary addition to the existing research that explores CLT mechanisms with a capacity view (Yan et al., 2016). Furthermore, the introduction of control construct echoes prior studies that investigate the influence of resource scarcity reminder (Goldsmith et al., 2020), (dis)orderliness (Li et al., 2019), and low (vs. high) visibility caused by air pollution (Ding et al., 2020) on construal level. Second, given that probing mechanism underlying CLT not only gives a clearer and more in-depth understanding of the theory but also provides directions to explore what boundary factors influence CLT, this research introduces LOC and testifies its moderating role. The results demonstrate the reversal of CLT prediction when one’s LOC is external, extending prior research on CLT reversal (Kyung et al., 2014; Yan et al., 2016). Overall, the general pattern of findings echoes other studies on personal factors that moderate distance-construal relationships (Steidle et al., 2011; Wan and Agrawal, 2011), enriching studies on boundaries and reversals of CLT. Last but not the least, the research has the potential implication to unite moderators of CLT from prior literature under the umbrella of control. For instance, previous literature on CLT moderators reveals external factors such as novelty and ambient darkness (Förster et al., 2009; Steidle et al., 2011), as well as personal traits such as self-control capability, involvement, self-construal, and knowledge (Lee et al., 2011; Wan and Agrawal, 2011; Bruyneel and Dewitte, 2012; Kyung et al., 2014). Based on the control’s definition, uncertainty caused by factors such as novelty and darkness could reduce individuals’ control perception. Self-control capability, involvement, self-construal, and knowledge might impact individuals’ personal mastery and perceived constraints, consequently influencing their perceived control. The new framework for conceptualizing the mechanism underlying CLT is also generative for future research because any context that influences control perception shall play moderating role on distance-construal relationship.

For control theory, the findings make another three noteworthy contributions. Primarily, the research investigates a new determinant of perceived control—psychological distance. It serves as an individual-level factor that interacts with environmental affordance (Wu and Lin, 2012) to influence perceived control, conflicting with other social and cultural factors (Sastry and Ross, 1998) that are interacting with the external environment at the group level. Another contribution relates to outcomes of perceived control. Differing from prior literature that examines physical phenomena or psychological well-being due to control (Abramson et al., 1978; Karasek, 1990; Perry and Morris, 2005; Cutright and Samper, 2014), this research innovatively measures CL as a dependent variable, proving that high (vs. low) perceived control triggers concrete (vs. abstract) CL. Thus, the findings extend the strand of research on how control impacts humans cognitively, echoing the study of Zhou et al. (2012). A third input involves the control compensatory theory (Inesi et al., 2011). On one hand, it shows that high CL could be considered a manifestation of the structure-seeking process to regain control, adding to the existing literature on structure-seeking (Cutright, 2012; Cutright et al., 2013). On the other hand, the results indicate the possibility of another strategy when control is deprived, which is to engage in abstract thinking. This broadens the control compensatory strategy sets that comprise seeking structured consumption, relying on superstitious rituals, seeking status, supporting authoritative governments or political leaders, etc. (Keinan, 2002; Kay et al., 2008, 2010; Inesi et al., 2011; Shepherd et al., 2011; Cutright, 2012).

Practically, the findings may prove valuable for public organizations and companies. It is relevant to policy-makers interested in dealing with CL-related issues. For example, facing the COVID-19 pandemic, civilians at the center are vulnerable to negative influences caused by even the most trivial information; therefore, it is very essential for policymakers to guide people to apply a high level of construal to process information in a more analytical and systematic way to ease anxiety and fear. Inspired by the findings, three steps can be taken. First, create population profiles based on chronic control belief (i.e., LOC) information, which might help with making communication strategies group by group. Second, find out people whose LOC are external and make targeted efforts to persuade them to temporarily believe outcomes should be determined by what people do. Third, deliver clear and persuasive messages to arouse people’s motivation to pursue control, aiming to facilitate their high CL. As for companies, it is suggested that, when making communication/marketing strategies, they should consider whether it directly impacts consumers’ perceived control or not. For example, it is popular to put time limits/quantity limits/identity limits in promotion so that psychological proximity is made salient and can stimulate consumers’ concrete CL to increase their purchasing intention. Yet, implied by the findings that perceived control mediates the distance-construal relationship, if other situational factors reduce or compensate consumers’ control feeling, the effectiveness of marketing strategies aimed at evoking particular CL might be weakened or even dominated. Therefore, marketing managers should caution “boomerang effect” or “invalid expenditure” of marketing invests that are due to the interplay of marketing tactics and situational control factor (e.g., devices consumers use for shopping). Furthermore, marketing tactics’ effectiveness might reverse due to LOC. It is advised that companies should pay attention to consumers’ control belief, create consumer profiles concerning LOC information, and position their marketing effort with certain types of LOC depending on precise cases.

With respect to limitations and future research, several points are noteworthy. First, of the four dimensions of psychological distance, social, spatial, and hypothetical distances were examined in this current research, except temporal distance. Future research shall design experiments by differently manipulating temporal distance to test the validity of the findings. With regard to the situation involving temporal distance, one might claim that they feel more controllable over outcomes of a distant exam 1-month later than that of a proximal exam tomorrow. The link between temporal distance and perceived control might be moderated by individual traits such as planning habit. Hence, the proposed control-based mechanism underlying effect of temporal distance on the construal level might be more complex, and it is recommended to investigate the moderating role of planning habits on the distance-control link. Also, CLT states a bilateral relationship between psychological distance and CL (Bar-Anan et al., 2006; Liberman et al., 2007); yet, this current research is focused on the effect of psychological distance on CL. Future research should examine whether the proposed control-based mechanism route still works for construal-distance relationship. Correspondingly, the reversal of construal-distance association should be explored with LOC as a moderator to enrich relevant research by Kyung et al. (2014). Moreover, deriving from the theorizing, one can probe the effect of overconfidence on CLT, and it might turn out to be another personal trait that influences CLT prediction. In addition, this current research is heavily contingent upon control-related constructs, but the control literature suggests cultural differences for control-related constructs (i.e., LOC) (Sastry and Ross, 1998; Zhou et al., 2012). Even though the authors recruit western participants from the Prolific platform in study 1a and study 1b and eastern participants from the university in study 2, implying that the mediating role of perceived control for distance-construal effect shall not be culturally different, it would still be critical to consider whether there are cultural differences or not for the moderating role of LOC in the future. Lastly, this current article is just a trial to unpack the black box underlying distance-construal association with a motivational view. Future work could explore alternative motivation-viewed and/or capacity-viewed mediating factors to provide a more nuanced understanding of the CLT.

## Data availability statement

The original contributions presented in this study are included in the article/supplementary material, further inquiries can be directed to the corresponding author.

## Ethics statement

Ethical review and approval was not required for the study on human participants in accordance with the local legislation and institutional requirements. Written informed consent from the patients/participants or patients/participants legal guardian/next of kin was not required to participate in this study in accordance with the national legislation and the institutional requirements.

## Author contributions

H-DH made substantial contribution to the conception or design of the work. QZ made substantial contribution to conception of the work, as well as acquisition, analysis, and interpretation of data for the work. Both authors contributed to the article and approved the submitted version.
